# Factor analysis, emotional experience and behavioral feedback of contemporary Chinese youth participating in short-term monasticism: a qualitative study based on the online question-and-answer community Zhihu

**DOI:** 10.3389/fpsyg.2025.1492692

**Published:** 2025-03-28

**Authors:** Chao Liu, Li-Jen Lin, Hao Chen, Thu-Hua Liu, Huang-Li Lin, Wen-Ko Chiou

**Affiliations:** ^1^School of Journalism and Communication, Hua Qiao University, Xiamen, China; ^2^Business Analytics Research Center, Chang Gung University, Taoyuan, Taiwan; ^3^General Education Center, Ming Chi University of Technology, New Taipei, Taiwan; ^4^Mindfulness Meditation Center, Ming Chi University of Technology, New Taipei, Taiwan; ^5^School of Film Television and Communication, Xiamen University of Technology, Xiamen, China; ^6^Department of Industrial Design, Ming Chi University of Technology, New Taipei, Taiwan; ^7^Department of Psychiatry, Chang Gung Memorial Hospital, Taoyuan, Taiwan; ^8^Department of Industrial Design, Chang Gung University, Taoyuan, Taiwan

**Keywords:** short-term monasticism, youth spiritual practice, religious psychology, cultural inheritance, qualitative research

## Abstract

**Introduction:**

This study explores the psychological motivations, emotional experiences, and behavioral feedback of contemporary Chinese youth participating in short-term monasticism. Despite the rising popularity of these practices as a means of stress relief and spiritual exploration, limited research has addressed their socio-cultural and psychological implications in the context of modern Chinese society.

**Methods:**

Data were collected from Zhihu, a leading online Q&A community in China, using Python web crawler technology. Relevant discussions and comments were systematically selected based on predefined criteria. The collected qualitative data were analyzed using NVivo software, with grounded theory guiding the open, axial, and selective coding processes to identify key themes and categories.

**Results:**

The analysis revealed that short-term monasticism functions as both a stress relief mechanism and a catalyst for spiritual growth and value formation. Key themes identified include the pursuit of inner peace, spiritual exploration, self-improvement, and a deepened engagement with traditional culture. Participants reported enhanced emotional regulation, self-reflection, and an increased appreciation for their cultural heritage.

**Discussion:**

The findings provide new insights into the role of short-term monastic practices in addressing mental health challenges and fostering personal and spiritual development among Chinese youth. These results underscore the potential of integrating traditional spiritual practices with contemporary mental health interventions, offering a holistic approach to enhancing well-being. Future research should explore the long-term effects of these practices and examine their applicability across different cultural contexts.

## 1 Introduction

The importance of mindfulness as an intervention in psychological research has surged in recent years, garnering substantial attention for its effects on mental wellbeing, emotional regulation, and stress reduction (Liu et al., 2020b, 2021, 2022a, 2023; Chiou et al., 2021). However, a significant gap remains in current literature regarding the origins of mindfulness. While much of the contemporary research presents mindfulness as a secular, non-religious practice, its roots in Buddhist monastic traditions are frequently overlooked or deliberately underemphasized (Purser, 2021). This trend toward the de-religionization of mindfulness can obscure a comprehensive understanding of the practice’s spiritual dimensions and original purpose, which inherently involve ethical and philosophical components drawn from Buddhism (Chiou et al., 2022; Kabat-Zinn, 2003).

Given that mindfulness originated as a Buddhist practice aimed at cultivating ethical behavior, spiritual growth, and self-awareness, the disconnect between its religious roots and modern psychological research raises concerns about the full understanding of its transformative potential (Galante et al., 2023). Research focusing on the practice of mindfulness meditation that incorporates its religious and spiritual origins remains relatively sparse, limiting our ability to explore how individuals can utilize this deeply rooted practice for both psychological and spiritual wellbeing (Levin, 2023). This lack of integration may hinder the depth of mindfulness’s application in therapeutic settings, where acknowledging its religious context could enhance participants’ experience, particularly in cultures that value spiritual traditions. In recent years, national surveys and trend analyses have revealed a marked increase in the participation of Chinese youth in short-term monastic activities. For example, data from recent studies indicate that participation rates have grown by an average of 15% annually over the past decade, reflecting a significant shift in how young people seek stress relief, spiritual growth, and cultural reconnection (Liu et al., 2021). This surge underscores the rising appeal of short-term monasticism as a viable means for emotional and psychological support in modern society. By highlighting these trends, we can better contextualize the relevance and scope of this study, emphasizing the importance of understanding such phenomena within today’s rapidly changing cultural landscape (Liu et al., 2020b).

Mindfulness, as a psychological practice, has deep roots in Buddhist monastic traditions. Originally cultivated in Buddhist meditation practices, mindfulness has evolved into a modern psychological tool used for enhancing wellbeing and managing stress. The Buddhist concept of mindfulness, or “sati,” involves paying attention to the present moment with awareness and acceptance, which is fundamental in practices like meditation (Liu et al., 2023). Over time, this practice has been integrated into contemporary psychology, particularly in therapeutic settings such as Mindfulness-Based Stress Reduction (MBSR) and Mindfulness-Based Cognitive Therapy (MBCT). These modern adaptations of mindfulness have been shown to have a positive impact on mental health, including reducing symptoms of anxiety, depression, and stress (Chen et al., 2023). By expanding on the relationship between Buddhist traditions and modern psychology, we can better understand the theoretical foundation of mindfulness and its relevance to the study of short-term monasticism. This foundation will help clarify how the ancient practice of mindfulness, rooted in spiritual and philosophical teachings, intersects with contemporary psychological interventions aimed at improving mental wellbeing (Chiou et al., 2022).

This study seeks to address this gap by exploring the psychological motivation, emotional experience, and behavioral feedback of contemporary Chinese youth participating in short-term monastic retreats. The Buddhist origins of mindfulness and their relevance to modern psychological and therapeutic practices are central to this discussion (Liu et al., 2022b). By examining short-term monasticism, which integrates both the spiritual and religious dimensions of mindfulness, we aim to offer insights into how young people navigate stress, seek spiritual fulfillment, and engage with traditional cultural values in a rapidly modernizing society (Kristeller and Rapgay, 2013).

Using the popular online community Zhihu as a source of data, this study employs qualitative methods to uncover the lived experiences of young people engaged in these retreats. Through the lens of religious coping theory (Pargament et al., 2000), we examine how short-term monastic life functions as a mechanism for stress relief, spiritual exploration, and identity formation. This theoretical framework allows us to explore not only the psychological impacts of monasticism but also its broader cultural and religious implications for the spiritual lives of youth in contemporary China (Dolcos et al., 2021).

This study is anchored in two primary theoretical frameworks that elucidate the psychological and spiritual dimensions of short-term monasticism. First, Religious Coping Theory (Pargament et al., 2000) posits that individuals draw on their religious beliefs and practices to manage stress and navigate life’s challenges. In the context of short-term monasticism, this theory helps explain how young people utilize structured religious practices—such as meditation, ritual observance, and community engagement—to alleviate stress, achieve emotional regulation, and foster spiritual growth (Zheng et al., 2025). In addition, Mindfulness Theory offers a complementary perspective by emphasizing present-moment awareness and non-judgmental acceptance as key components of psychological wellbeing (Wang et al., 2025). Originating from Buddhist meditation practices, Mindfulness Theory has been widely adopted in modern therapeutic interventions such as Mindfulness-Based Stress Reduction (MBSR) and Mindfulness-Based Cognitive Therapy (MBCT) (Liu et al., 2025). By integrating Mindfulness Theory with Religious Coping Theory, this study gains a more comprehensive understanding of how the ancient practices of mindfulness not only mitigate stress but also enhance self-awareness and spiritual fulfillment among contemporary Chinese youth. By integrating the religious context of mindfulness back into the discussion, this study not only fills a significant theoretical gap but also enriches the practical understanding of how mindfulness, when practiced within its original Buddhist framework, can serve as a powerful tool for both psychological and spiritual wellbeing. Furthermore, it contributes to the broader discourse on the role of religion and spirituality in mental health interventions, particularly in societies where traditional spiritual practices still hold significant cultural relevance.

## 2 Materials and methods

### 2.1 Data sources and collection

This study aims to deeply explore the motivations, emotional experiences, and behavioral feedback of contemporary Chinese youth participating in short-term monastic retreats, with a particular focus on qualitative data collected from the online Q&A community Zhihu. Zhihu, as a leading knowledge-sharing platform in China, attracts a broad user base, including many young people who are interested in or have experience with short-term monastic retreats, making it an ideal data source for this study. The primary data collection method used in this study was Python web scraping, which was employed to analyze discussions, Q&A sessions, and personal experience sharing about short-term monastic retreats on Zhihu. The research team systematically searched for posts and comments containing keywords such as “short-term monastic retreat,” “Buddhist practice,” and “youth and religion,” ensuring a wide range of perspectives and experiences were covered. The data collection period extended up to December 2023 to capture relevant discussions and trends in recent years. Out of the initially collected 5,000 relevant discussions and comments, 350 were ultimately included in the analysis after applying strict selection criteria. The selection criteria were as follows: (1) Explicit Expression of Short-term Monastic Experience or Strong Interest: Only discussions and comments that clearly described personal short-term monastic experiences or expressed strong interest in short-term monastic retreats were included. (2) Detailed Descriptions: Priority was given to entries that provided detailed personal experiences, emotional reactions, and reflections on the impact of short-term monastic life, excluding those that were vague or unrelated to the research topic. (3) Exclusion of Duplicate Content: Highly repetitive or similar discussions and comments were excluded to ensure the diversity and uniqueness of the samples. After the screening process, the final sample consisted of 350 entries. These samples provided a rich data foundation for subsequent qualitative coding and analysis. The collected data were processed and analyzed using NVivo 12 software. The research team first coded the data, identifying key themes and concepts, and then compared and merged similar responses and comments to form a comprehensive description of the motivations, emotional experiences, and behavioral feedback of young people participating in short-term monastic retreats. Additionally, by cross-verifying responses and comments from different users, the reliability and representativeness of the findings were enhanced (Strauss and Corbin, 1998).

### 2.2 Data source and collection—data analysis

**Figure 1:**
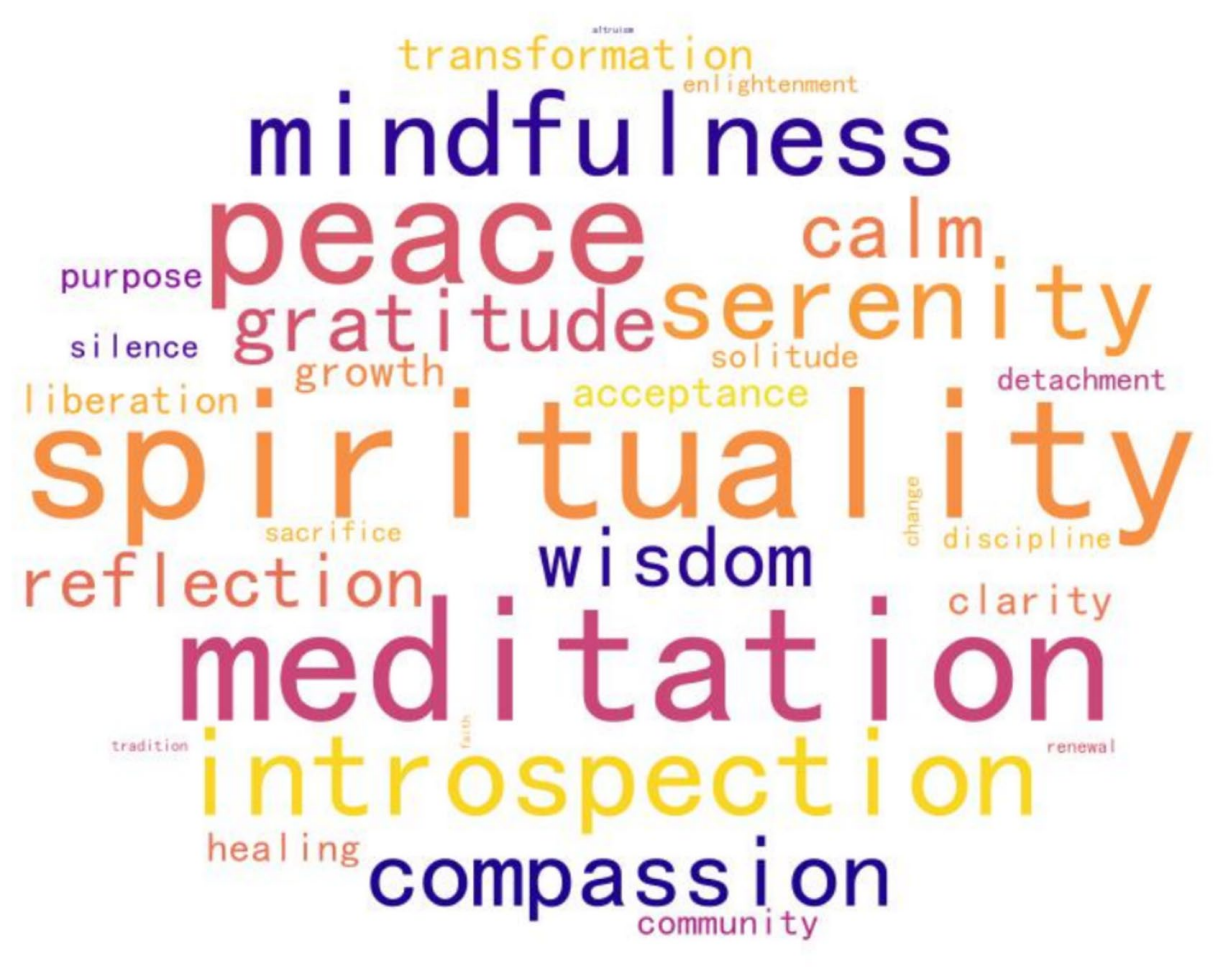
High frequency word cloud.

This study adopts qualitative research methods, especially grounded theory-based research strategies, to explore the factors analysis, emotional experience, and behavioral feedback of contemporary Chinese young people participating in short-term monasticism (Martin et al., 2018; Turner and Astin, 2021). Before starting to write the code, the text data is first preprocessed using NVivo 12 software. Through the word frequency analysis function, I generated a word cloud of high-frequency words. This step removes extraneous words, resulting in a cloud of high-frequency words (see Figure 1).

#### 2.2.1 Open coding

In the process of data collection, we first carried out open coding. This step involves a word-for-word analysis of discussions, questions, and answers, and sharing of personal experiences about short-term monasticism on Zhihu to identify emerging keywords, concepts, and categories. Through careful reading and repeated review, we labeled primary codes related to short-term monastic motivations, emotional experiences, and behavioral feedback, such as “seeking spiritual peace,” “inner exploration,” and “feelings of social isolation.” During this procedure, we mark the original text, resulting in 50 initial nodes (a1-a50). By analyzing and comparing these nodes, we consolidate some and reclassify them into 20 preliminary categories (A1-A20) (Table 1).

**Table 1:** Open coding (excerpt).

Original texts (excerpt)	Free node (excerpt)
I felt a lot of stress and confusion in my life and wanted to find a way to cleanse my mind (a1)	a1 Seek spiritual purification
Becoming a monk for a short period allowed me to gain an in-depth understanding of Buddhist culture, which was an unforgettable spiritual journey (a2)	a2 Learn more about Buddhist culture
My time in the temple allowed me to get away from the hustle and bustle of the city and feel real peace (a3)	a3 Find inner peace
I had new thoughts about my lifestyle and values and began to pay more attention to my spiritual life (a4)	a4 Self-reflection and value change
The communication with other monks made me realize the importance of serving others, and I was more willing to participate in public welfare activities after returning to society (a5)	a5 Service to others and community participation
During my monasticism, I practiced daily meditation, which helped me reduce anxiety and stress (a6)	a6 Meditation for stress reduction
Through this experience, I learned how to control my emotions and have more patience and acceptance for life (a7)	a7 Emotional control and life acceptance
I realized that material pursuit does not bring long-term happiness, but inner satisfaction is more important (a8)	a8 Reflection on inner satisfaction and material pursuit

#### 2.2.2 Axial coding and selective coding

In the next axial coding phase, we begin to connect the concepts identified in the open coding phase to form a more complex classification system. This includes the exploration of relationships between primary codes, such as causality, conditional relationships, and so on. Through this process, we were able to construct a central theme and dimension around short-term monasticism, further refining the research framework.

In the selective coding phase, we combined all the data and coding to identify the core category of the study, which is “seeking peace of mind and self-actualization.” Around this core category, we integrate related sub-categories and codes to construct a complete theoretical framework for the impact of short-term monastic experiences on young people’s spiritual lives and social behavior.

After a detailed examination and synthesis of the 20 initial categories using open coding and axial coding, we established 8 main categories (B1–B8). This process led to the identification of 3 basic categories (C1–C3).

#### 2.2.3 Coding saturation test

To ensure the integrity of the coding and the reliability of the research results, we conducted a coding saturation test. We confirm code saturation by constantly reviewing collected data and existing codes until new data no longer generates new code or modifies existing code. This shows that the data we have collected is sufficient to cover all aspects of the research problem, providing a solid foundation for further analysis and theoretical construction, and the three-level coding system is shown in Table 2.

**Table 2:** The three-level coding system.

Open coding	Axial coding	Selective coding
Initial category	Main category	Core category
A1 Stress reduction and resilience	B1 Seeking inner peace and spiritual fulfillment	C1 Reasons contemporary Chinese youth participate in short-term monastic life
A2 Spiritual exploration and self-improvement		
A3 Escape from reality and reflect on life		
A4 Learn more about Buddhist culture	B2 Interest in Buddhist culture and philosophy	
A5 Experience religious life		
A6 The pursuit of spiritual purification and promotion		
A7 Promote and experience traditional culture	B3 Respect and legacy for traditional culture	
A8 Searching for roots and self-identity		
A9 Sense of social and cultural responsibility		
A10 Inner peace and serenity	B4 Positive emotional experience	C2 Emotional experiences of contemporary Chinese youth in short-term monastic life
A11 Selflessness and flow		
A12 Subjective wellbeing		
A13 Feelings of loneliness and isolation	B5 Negative emotional experience	
A14 Feelings of frustration and maladjustment		
A15 Anxiety and uncertainty		
A16 Emphasizing a simplified lifestyle	B6 Changes in lifestyle	C3 Behavioral feedback of contemporary Chinese youth in short-term monastic life
A17 Increasing meditation and self-reflection habits		
A18 Improving dietary and health habits		
A19 Enhancing tolerance and gratitude	B7 Improvement in social interaction and interpersonal relationships	
A20 Enhancing sense of social responsibility		
A21 Improving intimate family relationships		
A22 Deepening understanding of religious or spiritual philosophy	B8 Deepening spiritual beliefs and values	
A23 Valuing inner worth and personal growth		
A24 Forming prosocial and altruistic behavior		

## 3 Results

### 3.1 Reasons for contemporary Chinese young people to participate in short-term monasticism

#### 3.1.1 Seek peace of mind and spiritual satisfaction

This study explores the psychological motivation and emotional experience of young people who participate in short-term monasticism and reveals the complex psychological needs and spiritual pursuits behind this phenomenon. The motivation and experience of seeking peace of mind and spiritual satisfaction, stress reduction and resilience enhancement, spiritual exploration and self-improvement, escapism, and reflection on life together constitute the main reasons for young people choose short-term monasticism.

##### 3.1.1.1 Decompression and resilience enhancement

In the fast-paced modern life, young people face pressure from many aspects, including career development, interpersonal relationships, and social expectations. Short-term monasticism provides a unique environment that allows them to temporarily detach from the stressors of everyday life. “During my days at the monastery, the daily meditation and simple living allowed me to escape the hustle and bustle of the outside world, and I felt more relaxed than ever.” This experience not only provides them with an immediate stress reduction effect but also enhances their ability to cope with life’s challenges in the long term by increasing their resilience.

##### 3.1.1.2 Spiritual exploration and self-improvement

Short-term monasticism is also seen as a way of spiritual exploration and self-improvement. “Living in a monastery has allowed me to gain insight into the wisdom of Buddhism and practice, which has been a valuable journey of spiritual exploration and self-improvement.” By learning Buddhist teachings and participating in spiritual practice, young people not only gain spiritual enrichment and growth but also gain self-knowledge and spiritual values.

##### 3.1.1.3 Escapism and reflection on life

In addition, short-term monasticism is also seen by some young people as an opportunity to escape reality and reflect deeply on life. “During this time, I was able to let go of my roles and responsibilities in real life and have more space to think about the meaning and direction of my life.” This experience of stepping away from everyday life not only provides a temporary escape, but also inspires deep reflection on life, values, and future directions.

#### 3.1.2 Interest in Buddhist culture and philosophy

By exploring young people’s interest in Buddhist culture and philosophy, this study reveals the profound impact of short-term monasticism on them. An in-depth understanding of Buddhist culture, the experience of religious life, and the pursuit of spiritual purification and improvement not only enrich the spiritual world of young people but also provide valuable opportunities for their personal growth and self-knowledge.

##### 3.1.2.1 In-depth understanding of Buddhist culture

Young people often participate in short-term monasticism out of deep interest and curiosity in Buddhist culture. “I am deeply interested in the aesthetics, history, and philosophy of Buddhism, and being a monk for a short period allows me to get closer and understand this ancient religious culture in greater depth.” This in-depth understanding not only increased their knowledge base but also gave them a more comprehensive and profound understanding of Buddhist culture.

##### 3.1.2.2 Experience religious life

Short-term monasticism offers a unique opportunity for young people to experience first-hand the religious life of Buddhism. “Living in a monastery and following the daily routines and practices of monks, I experienced a calmness and purity that was completely different from everyday life.” This experience is not only an escape from everyday life but also a spiritual baptism and rebirth.

##### 3.1.2.3 Pursue spiritual purification and enhancement

Many young people participate in short-term monastics out of the pursuit of spiritual purification and self-improvement. “In the busy and impetuous modern life, I longed to find a way to purify my spirit. By becoming a monk for a short time, I felt spiritual sublimation and inner peace.” This pursuit not only reflects young people’s deep thinking on spiritual values and the meaning of life but also reflects their desire for inner growth and spiritual improvement.

#### 3.1.3 Respect and inheritance of traditional culture

This study explores the psychological motivation of young people to participate in short-term monasticism and deeply understands their emotional experience of respecting and inheriting traditional culture. Carrying forward and experiencing traditional culture, searching for roots and self-identity, as well as social and cultural responsibility not only reflect young people’s deep affection for traditional culture but also show their positive role in inheriting and developing traditional Chinese culture.

##### 3.1.3.1 Promote and experience traditional culture

Many young people choose to become monks for a short period, out of the desire to promote and experience traditional Chinese culture. “Short-term monasticism is not only for personal practice but also an opportunity to deeply understand and experience China’s time-honored Buddhist culture.” This experience enables them to have direct contact with an in-depth understanding of the essence of Buddhist culture, enhancing their awareness and pride in the country’s traditional culture.

##### 3.1.3.2 Search for roots and self-identity

Short-term monasticism has also become a way for young people to find their roots and explore self-identity. “By learning Buddhist teachings and participating in monastic life, I began to reflect on my lifestyle and values and find a deeper self-identity.” This process not only helps them to establish a connection with traditional culture but also promotes a deep understanding of personal identity and the meaning of life.

##### 3.1.3.3 Social and cultural responsibility

In addition, young people who participate in short-term monascations often express a strong sense of social and cultural responsibility. “I hope that through my experiences and actions, I can convey the wisdom of Buddhist culture and the value of traditional Chinese culture to more people.” This sense of responsibility drives them not only to pay attention to personal spiritual growth but also to the protection, inheritance, and promotion of traditional culture, hoping to contribute to the maintenance and development of national culture.

### 3.2 The emotional experience of contemporary Chinese young people participating in short-term monasticism

#### 3.2.1 Positive emotional experience

This study reveals the positive emotional effects of short-term monasticism on contemporary Chinese youth, including inner peace and tranquility, egoism and flow, and subjective wellbeing. These experiences not only enrich the spiritual life of young people but also promote their progress in personal growth and spiritual exploration.

##### 3.2.1.1 Inner peace and tranquility

Many young people seek inner peace and tranquility by becoming monks for short periods. “In the hustle and bustle of my life, becoming a monk for a short period became a way for me to find inner peace. My practice in the temple has made me feel more peaceful than ever before.” This experience is not only a temporary escape from the stresses of daily life but also a spiritual purification and reset, providing participants with an inner space of peace and tranquility.

##### 3.2.1.2 No self and flow

Short-term monascations also provide young people with the opportunity to enter a state of flow, which is an experience of being completely immersed in an activity to the point of self-abandonment. Activities such as meditation, chanting, and meditation allow participants to fully focus on the present moment and experience a state of flow. “When I am in meditation, completely immersed in every breath, everything outside seems to disappear for a moment, and this concentrated experience gives me a real sense of release.” This depth of concentration not only enhances their spiritual experience but also helps them to be more focused and productive in their daily lives.

##### 3.2.1.3 Subjective wellbeing

Short-term monasticism has an important impact on improving the subjective wellbeing of young people. By participating in monastic life and Buddhist practice, young people reassess what is true happiness in life. “Through this experience, I began to appreciate the little things in life more and my satisfaction with life has increased significantly.” This new perspective on life and an attitude of gratitude directly increased their happiness, making them more able to appreciate and value their current situation in life.

#### 3.2.2 Negative emotional experience

This study provides a more comprehensive understanding of this phenomenon by exploring the negative emotional experience of young people during the short-term monastic process. Negative emotional experiences, such as feelings of loneliness and isolation, frustration and maladjustment, and anxiety and uncertainty, are an inevitable part of the short-term monasticism process, but they also provide opportunities for self-reflection and growth.

##### 3.2.2.1 Loneliness and isolation

Many young people experience a profound sense of loneliness and isolation during their short monastic periods. “I felt more lonely in the monastery than ever before, separated from my familiar social environment and my friends and family.” This sense of loneliness stems not only from physical isolation but also from spiritual isolation, especially in the face of the great difference in the way of life of the Sangha.

##### 3.2.2.2 Frustration and inadaptability

Frustration and maladjustment are common challenges faced by many young people who participate in short-term monasticism. “It was very difficult for me to adjust to the temple rules and the pace of life, which made me feel deeply frustrated.” This frustration stems not only from unfamiliarity with the rules of Buddhist practice but also from the difficulty of quickly integrating into the monastic way of life.

##### 3.2.2.3 Anxiety and uncertainty

In the short-term monastic process, anxiety and uncertainty are also a problem that cannot be ignored. “I felt lost about the direction I was going in and wasn’t sure if this experience would help me find peace of mind.” This anxiety involves not only concerns about one’s future but also doubts about whether short-term monasticism will have the desired effect.

### 3.3 Behavioral feedback of contemporary Chinese young people participating in short-term monasticism

#### 3.3.1 Changes in lifestyle

This study reveals the profound impact of short-term monasticism on the daily life of young people in China through an in-depth analysis of their lifestyle changes after taking part in short-term monasticism. Changes such as a greater focus on minimalism, increased habits of meditation and self-reflection, and improved diet and health habits not only boosted participants’ mental and physical health but also reflected the positive role of short-term monastic experiences in guiding individual lifestyles.

##### 3.3.1.1 Pay more attention to simple life

Many young people, after a short period of monastic experience, begin to prefer a more minimalist lifestyle. “The short-term monastic experience made me aware of many unnecessary material pursuits in my life, and I began to try to reduce material consumption and focus on improving my inner spiritual life.” This transformation not only lightens the burden of life but also makes their lives more comfortable and fulfilling.

##### 3.3.1.2 Increase the habit of meditation and self-reflection

The experience of participating in short-term monastics has also encouraged young people to add meditation and self-reflection to their daily lives. “Daily meditation and self-reflection became a part of my life, which not only helped me maintain peace of mind but also made me more aware of myself and the world around me.” Through these habits, they can better manage emotions and stress while promoting personal spiritual growth.

##### 3.3.1.3 Improve diet and health habits

Short-term monasticism not only affects the spiritual life of young people but also promotes improvements in their diet and health habits. “The vegetarian life in the monastery allowed me to experience the benefits of a healthy diet, and when I returned to my daily life, I began to pay more attention to the health and balance of my diet.” This change not only improved their physical health but also increased their awareness of respect and gratitude for life.

#### 3.3.2 Improvement of social interaction and interpersonal relationship

This study reveals the positive effects of short-term monasticism on the social interaction and interpersonal relationships of contemporary Chinese young people, including the enhancement of tolerance and gratitude, the enhancement of social responsibility, and the improvement of family intimacy. These positive changes not only promote young people’s personal growth and mental health but also provide a solid foundation for their harmonious interaction with society and the family.

##### 3.3.2.1 Promote tolerance and gratitude

The short monastic experience made the participants more tolerant and grateful, and the enhancement of these qualities directly improved their interpersonal skills. The emphasis on compassion and tolerance in religious practice helps them to be more open to different people and perspectives in their daily lives. “Through my study and practice in the monastery, I learned how to truly understand and tolerate others, which not only changed the way I get along with my friends and colleagues but also made me gentler towards myself.” In addition, increased gratitude led participants to express gratitude to others more frequently, which enhanced positive feedback and interaction in social relationships.

##### 3.3.2.2 Enhance social responsibility

Young people who participate in short-term monasticism often report that the experience has enhanced their sense of social responsibility. “During my time as a monk, I deeply experienced the impact of individual actions on society and the environment, which inspired me to have a deep understanding of social responsibility.” This enhanced sense of social responsibility encourages them to participate more actively in public welfare activities and social services after returning to society and strive to contribute to social harmony and progress.

##### 3.3.2.3 Improving family intimacy

One of the most significant changes is the positive impact of short-term monasticism on family relationships. The patience, tolerance, and communication skills that the participants learned through the practice effectively improved their close relationships with their families. “After I came home, my relationship with my parents and siblings improved significantly. I learned how to listen better to their needs and be more willing to share my feelings and thoughts.” This improvement not only enhances the harmony of the family but also provides a solid foundation for the psychological security of the individual.

#### 3.3.3 Deepening of spiritual beliefs and values

This study explores the influence of short-term monasticism on the deepening of young people’s spiritual beliefs and values and reveals the positive effects of this experience on personal spiritual life and social behavior. A deeper understanding of religious or spiritual philosophy, an emphasis on inner values and personal growth, and a desire to form or strengthen service to others contribute to the spiritual maturity and value development of young people.

##### 3.3.3.1 Deepen your understanding of religion or spirituality

Short-term monasticism offers young people a unique opportunity to focus on spiritual and religious exploration outside of their daily lives. In the process, they are exposed to esoteric Buddhist teachings and meditation techniques, and these experiences help them to understand the nature of religion or spirituality at a deeper level. “Through daily meditation and scripture study, I gained a more intuitive sense of the core tenets of Buddhism and began to understand how they guide my personal life.” This deeper understanding allows young people to gain richer spiritual nourishment.

##### 3.3.3.2 Value intrinsic value and personal growth

The short-term monastic experience encourages young people to place greater emphasis on intrinsic values and personal growth. “I’ve come to realize that true happiness and fulfillment comes from inner peace and personal growth, rather than external material possessions and accomplishments.” This shift in perception not only changes their definition of success and happiness but also inspires them to pursue spiritual enrichment and self-improvement in their daily lives.

##### 3.3.3.3 Forming prosocial and altruistic behaviors

In addition, short-term monasticism also forms or reinforces young people’s desire to serve others. “My experience in the monastery has taught me the joy and value of serving others, and I hope to bring this spirit back to my daily life and work.” This desire to serve others is not only a practice of the Buddhist concept of compassion but also a manifestation of a shift in young people’s values toward a more social and altruistic approach.

## 4 Discussion

Based on Zhihu, China’s largest online question-and-answer community, this study explores in depth the motivations and experiences of contemporary Chinese young people who participate in short-term monasticism. Through a qualitative analysis of texts shared by participants in the community, we reveal the various psychological and social motivations for participating in short-term monasticism and the profound impact this experience has on individuals’ emotional experiences and behavioral patterns. In addition, the study explores how young people cope with the stress of life through various spiritual practices during the short-term monastic process and the positive contribution of these practices to their positive psychology.

### 4.1 Spiritual ascension

In this study, young people’s participation in short-term monasticism is mainly motivated by the need for personal spiritual growth, which is particularly prominent in contemporary Chinese society, where traditional spiritual and cultural pillars are challenged due to rapid economic development and the diversification of social values (Liu et al., 2020a). Short-term monasticism provides a unique environment in which young people can temporarily detach from material pursuits and focus on inner spiritual practice (Dorais and Gutierrez, 2021). This environment promoted deep self-reflection and spiritual exploration, and many participants reported that the experience helped them reevaluate their personal life goals and values (Bringmann et al., 2021a,b). Through daily chanting and meditation, individuals learn how to let go of complex thinking and find peace of mind (Jiwani et al., 2022). Although short-term monasticism is a traditional religious practice, its application in contemporary society shows the characteristics of modernity (Yaden et al., 2023). Young people try to apply the spiritual practice skills learned in practice to their daily lives to cope with the pressures and challenges of modern life (Bahadorani et al., 2021). Practical meditation in daily life helps individuals stay calm and focused at work and in interpersonal interactions (Tseng, 2022). Spiritual ascension is not limited to experiences during monasticism but also involves the integration of these spiritual practices into a broader life scenario (Silvestre-López, 2020). Research has also shown that spiritual uplift is often accompanied by a deepening sense of social responsibility (Bringmann et al., 2021a,b). Through the short-term monastic experience, young people become more concerned about social and environmental issues and change their behavior to reflect higher moral and ethical standards to some extent (Tsui et al., 2020). Monasticism makes individuals realize that every choice in life is related to the world around them, and individuals begin to pay more attention to ecological protection and social justice (Thirathamrongwee and Mongkolpradit, 2022).

### 4.2 Improvement of subjective wellbeing

The study found that participating in short-term monasticism not only affected participants’ spiritual and psychological levels but also significantly improved their subjective wellbeing (Van Phong, 2022). The short monastic experience usually involves a series of religious activities and spiritual practices, such as meditation, chanting, and meditation, which have been shown to reduce stress and promote a sense of inner peace (Thanh et al., 2023b). By participating in short-term monasticism, the individual learns how to manage emotions and stress, which allows the individual to feel more calm and content in daily life (Singh and Kumar, 2022). This inner sense of peace leads to a deeper sense of satisfaction, which boosts their overall happiness. Although short-term monasticism may involve a degree of loneliness and isolation, it also offers the opportunity to form deep connections with fellow travelers (Thanissaro and Lincoln, 2023). This social connection based on shared spiritual pursuit strengthens the sense of belonging and is an important factor in enhancing subjective wellbeing (Ardelt et al., 2023). During the monastic period, the individual connects with many like-minded people, and this deep social interaction enhances my sense of belonging and happiness (Qin and Song, 2020). This social support is not only important during the monastic period, but these social ties continue to play a supportive role after returning to daily life (Udayanga, 2020). Participating in short-term monasticism also encourages young people to reflect and affirm their values, which is one of the key factors in improving subjective wellbeing (Huong and Phan, 2023). Short-term monasticism provides a unique environment and opportunity for participants to explore the meaning and purpose of life (Thanh et al., 2023a). Monasticism gives individuals time to think about the true meaning of life and redefine their values and life goals. Having a clear sense of purpose and meaning in life is recognized in psychological research as an important source of happiness (Nguyet, 2021).

### 4.3 Improvement of flow experience

We found that participants in the short-term monastic process often experienced a state of flow, which is a state of total immersion and high concentration that they achieve in their spiritual practice (Bronkhorst, 2020). Flow not only enhances their happiness but also promotes personal psychological growth and self-transcendence. Flow is a concept developed by psychologist Csikszentmihalyi, which refers to the experience of being completely engaged in an activity, feeling fun and satisfaction, and losing a sense of time (Matko et al., 2021). In the context of short-term monasticism, the flow of the mind often occurs in deep meditation, chanting, or other religious rituals (Gao et al., 2023). In the practice of deep meditation, the individual often enters a state of total self-oblivion, perceiving only the present moment, and that calmness and concentration allow the individual to experience great satisfaction and happiness (Church et al., 2022). The flow state not only improves the quality of young people’s experiences during monasticism but also has a positive impact on their mental health. Studies have shown that people who experience flow regularly are psychologically healthier, with lower feelings of stress and a greater sense of wellbeing (Harvey, 2022). Through continuous practice and meditation, Flow helps individuals overcome the anxiety and stress of daily life, allowing them to achieve true spiritual freedom (Herbert and Harmat, 2022).

In addition, flow experiences show significant value in promoting self-knowledge and personal growth. In a short-term monastic environment, young people deepen their understanding of self and life through flow experience, promoting self-improvement and spiritual maturity (Cooper et al., 2021). Flow is not only a pleasurable experience, it is also an important means of self-exploration and growth. In those moments of immersion, individuals gain a deeper insight into themselves and the world (Divino, 2023).

### 4.4 Improvement of resilience

Resilience, or resilience, refers to an individual’s ability to adapt, recover, or grow effectively in the face of stress, difficulty, or adversity (Grant, 2020). Through qualitative data analysis, we found that short-term monasticism not only helps young people face the challenges of life but also enhances their ability to recover and grow in the face of adversity (Falk, 2021). During the short monastic period, participants learned to better control their emotions and adjust their mindsets through spiritual practices such as meditation, chanting, and meditation (Dorais, 2021). These skills are an important part of resilience. The practice of monasticism teaches individuals how to remain calm in the face of stress, and this ability helps them face the challenges of work and relationships more calmly when they return to daily life (Phyu and Han, 2021). This suggests that spiritual practice is not only a religious ritual but also an effective way to promote mental health and resilience. Social support is another key factor in promoting resilience (Dorais and Gutierrez, 2021). During the short time away from home, participants developed deep connections with other monks and formed a supportive community (Cavaliere, 2021). This social support network provides emotional comfort and practical help. The deep communication with other practitioners enhanced my sense of social support, which made me no longer feel alone in the face of personal problems (Bormolini et al., 2022). Through this community support, young people’s ability to cope with adversity is significantly strengthened (Gajaweera and DeAngelo, 2021). Short-term monasticism also encourages participants to explore and affirm the spiritual meaning of life, which is another important way to increase resilience (Cook, 2021). A deep understanding of the meaning of life can enhance an individual’s psychological resilience in the face of difficulties (McManus, 2024). By understanding and practicing the teachings of Buddhism, individuals find deep meaning in life, and this discovery makes them more empowered to face life’s challenges. A clear sense of purpose and meaning in life helps young people maintain a positive attitude and resilience in the face of difficulties (Cassaniti, 2021).

### 4.5 Enhancement of tolerance

The promotion of tolerance is an important theme, especially as young people experience spiritual cultivation and self-improvement through short-term monasticism (Rahmani and Busro, 2023). Tolerance is not only an ability to accept different opinions and behaviors but also an embodiment of inner psychological maturity and spiritual growth (Guidotti et al., 2023). Short-term monascations provide a unique environment for participants to not only learn deeply about the Buddhist teachings of compassion and tolerance but also to experience and implement them in their daily practice (Lindahl et al., 2023). During the practice, the individual deeply appreciates the power of compassion and tolerance. When they begin to view others with this mindset, they find that their judgments and attitudes toward those around them become softer (Jagendal, 2022). This change not only improved their mental health but also their relationships. With the increase in tolerance, the social behavior of young people has also changed significantly (Park and Pinel, 2023). They are more inclined to interact with others in non-critical and supportive ways, and this change is seen both on social networks and in everyday life (Choe and O’Regan, 2020). By practicing tolerance, I can deal with conflict and social challenges more calmly and with less emotion. This tolerant attitude enables them to communicate and solve problems more effectively in society, promoting more harmonious social relations (Yi, 2022). Promoting tolerance not only affects individuals’ mental health and social abilities but also has a profound impact on the sociocultural environment (Galante et al., 2023). Increased tolerance contributes to a more open and inclusive society, where individuals begin to recognize that everyone has their lifestyle and choices, and this recognition allows them to be more open to different cultures and perspectives (Jijina and Biswas, 2022). This social effect is particularly significant in modern societies with rapid growth in diversity, helping to reduce social conflicts and improve public wellbeing (Shulman, 2021).

### 4.6 The improvement of gratitude

We observed a significant increase in feelings of gratitude among young participants, a change that not only enhanced their wellbeing but also improved their social interactions (Khan et al., 2021). Through daily meditation, chanting, and other religious rituals, young people experience the importance of letting go of the self and feeling the gifts in life (Medvedev et al., 2022). These practices reinforce their appreciation for the small things in life, thereby fostering a sustained mindset of gratitude (Marasinghe et al., 2021). During this time, individuals learn to cherish everything around them, whether it is simple interaction with people or small natural favors, they feel very grateful (Lau, 2022). This feeling of gratitude enables them to have a more positive attitude in the face of the stresses and challenges of daily life. Psychological studies have shown that sustained gratitude is associated with higher life satisfaction and lower feelings of depression and anxiety (Chen et al., 2023). The practice of gratitude during the monasticism of young people helps them to build a healthier mental state. Through the practice of gratitude in the practice, the individual finds that his dissatisfaction with life and complaints are significantly reduced, and his mood becomes more cheerful (Stang, 2022). This psychological transformation not only improved their emotional state but also enhanced their ability to deal with personal relationships and social pressures. The increase in gratitude also promotes positive interactions among young people in social relationships (Jeglič, 2022). They are more inclined to express gratitude and appreciation, and these behaviors enhance relationships with others (Poceski, 2020). Individuals began to express gratitude more frequently to those around them, whether family, friends, or colleagues, and it made my relationships better. In this way, gratitude not only enhances their relationships but also lays the foundation for a more harmonious social environment (King, 2023).

### 4.7 Practical implications

The findings of this study have significant implications for future research and practical applications in the fields of mental health, policy-making, and spiritual practices. The positive psychological effects of short-term monasticism on youth suggest that this practice could be explored as a therapeutic intervention for young people dealing with high levels of stress, anxiety, and other mental health challenges. Integrating short-term monastic practices or mindfulness-based interventions into mental health care could offer a holistic approach to therapy, focusing on emotional regulation, mindfulness, and personal growth. Such interventions could be particularly beneficial in settings where traditional therapies may not be fully effective or accessible (Pang and Ruan, 2024).

In the context of psychological counseling, short-term monasticism could be used as an adjunctive treatment to help young people cultivate coping mechanisms, increase emotional resilience, and manage stress in healthier ways. Mental health professionals could incorporate mindfulness and meditation techniques derived from short-term monasticism into their therapeutic practices, helping clients develop better self-awareness and coping strategies (Pang and Hu, 2024).

Additionally, these findings have important implications for campus guidance programs. Given the increasing mental health challenges faced by university students, short-term monasticism or related mindfulness practices could be integrated into university wellness programs or counseling centers. Universities could offer mindfulness-based retreats or workshops that provide students with opportunities to engage in practices that enhance mental wellbeing, improve focus, and reduce stress (Pang and Zhang, 2024a). By incorporating these practices, institutions could provide students with valuable tools for managing academic and personal stress while promoting their overall mental health. Furthermore, workplace stress management could benefit from these findings. Many young professionals face high levels of stress and burnout in the workplace, and short-term monasticism could be adapted into workplace wellness programs. Offering mindfulness-based initiatives, such as meditation sessions or retreats, could help employees manage work-related stress, enhance job satisfaction, and improve overall mental health (Pang and Zhang, 2024a). This approach would contribute to creating healthier work environments, particularly in fast-paced and high-stress industries. Lastly, the study encourages further investigation into the long-term impacts of short-term monasticism, particularly through longitudinal research. Future studies could explore the enduring psychological, emotional, and behavioral benefits of these practices, providing a more comprehensive understanding of their lasting effects (Wang et al., 2024b). Moreover, it would be beneficial to examine how social support networks and individual characteristics such as personality, cultural background, and socio-economic status influence the effectiveness of short-term monasticism in promoting mental health. Such research could lead to more tailored interventions that take these factors into account (Wang et al., 2024a).

In summary, this research not only contributes to the academic understanding of short-term monasticism but also highlights its practical potential for improving the psychological wellbeing of youth in a rapidly modernizing society (Pang and Zhang, 2024b). The findings point to several areas where short-term monasticism and related mindfulness practices can be integrated into existing mental health care frameworks, policy development, and workplace wellness initiatives, ultimately enhancing the mental and emotional resilience of young people (Deng et al., 2024).

### 4.8 Research limitations and future research directions

Although this study provides preliminary insights into the motivations, emotional experiences, and behavioral feedback of contemporary Chinese youth participating in short-term monasticism, some limitations also point to possible directions for future research.

Limitation of data sources: While the study collected a substantial amount of data from the Zhihu platform, it is important to note that the sample may not fully represent the entire population of contemporary Chinese youth participating in short-term monastic activities. The participants in this study were primarily gathered from one online platform, which may introduce selection bias, as Zhihu users may not encompass the full demographic diversity of those engaging in short-term monasticism. To enhance the representativeness of future studies, data collection could be expanded to include other platforms, such as WeChat, Douban, or even offline sources, such as in-person interviews or surveys. This would help capture a more diverse range of perspectives and provide a more comprehensive understanding of the phenomenon.

Cultural and geographic differences: The study primarily focuses on Chinese youth, and while it provides valuable insights into the motivations and experiences of this demographic, it does not explore potential cultural differences across regions within China or between countries. The motivations and experiences of youth participating in short-term monasticism may vary significantly depending on cultural or regional contexts. For example, youth from rural areas or those influenced by different Buddhist traditions may have different experiences compared to their urban counterparts. Furthermore, cultural factors outside of China, such as in countries with strong Buddhist traditions like Thailand or Japan, may also lead to different outcomes. Therefore, future studies should consider cross-cultural comparisons to explore how cultural and regional differences may shape the participation in short-term monastic activities. This will provide a more comprehensive understanding of the phenomenon across diverse cultural contexts.

Gender and Age Differences: While this study provides valuable insights into youth participation in short-term monastic activities, the impact of gender and age on motivations, experiences, and behavioral feedback was not fully explored. Given that both gender and age may influence how individuals engage with short-term monasticism, future research should explicitly examine the roles these factors play in shaping motivations for participation, emotional experiences, and the psychological and behavioral outcomes. Understanding how gender and age interact with spiritual practices will provide a more nuanced perspective, particularly in relation to psychological and emotional responses. For instance, it is possible that younger participants may have different motivations or emotional experiences than older participants, and that gender could further shape these responses. Since these factors were not comprehensively addressed in this study, we recognize this as a limitation and encourage further investigation into the influence of gender and age in future research.

Role of Social Support: While this study highlights the positive psychological effects of short-term monasticism on youth, it does not fully address the role of social support, such as that provided by family, friends, or peers, in shaping these effects. Social support could play a significant role in enhancing or moderating the psychological benefits observed during short-term monastic experiences. However, since our study primarily focused on individual experiences, we did not collect comprehensive data on participants’ social networks or how these networks may influence their experiences and outcomes. Future research should explore the role of social support in greater detail, considering how it interacts with the monastic experience to affect emotional wellbeing, coping mechanisms, and overall outcomes. This will help further understand the dynamic between individual spiritual practices and the broader social context.

Long-term effects unknown: While this study primarily focuses on the immediate psychological effects of short-term monasticism, it does not address the long-term psychological and behavioral impacts of such experiences. The effects observed in this study may be transient, and the lasting benefits or challenges that participants experience once they reintegrate into their daily lives remain unknown. Future research should incorporate longitudinal studies to track the long-term changes in participants’ psychological, emotional, and behavioral outcomes. This would provide a more comprehensive understanding of how short-term monastic experiences influence individuals over time and whether the benefits observed are sustained or evolve after the initial experience.

Comparative studies: Similar studies were conducted in different religious contexts to compare the impact of short-term practice programs in religions such as Buddhism and Christianity on young people. Quantitative research: Using scale and statistical analysis methods, the psychological and social effects of short-term monasticism were quantitatively evaluated to enhance the objectivity and universality of the research.

## 5 Conclusion

This study explores the psychological motivation, emotional experience, and behavioral feedback of contemporary Chinese youth who participate in short-term monasticism. Studies have found that participating in short-term monasticism not only helps young people cope with the pressures of modern life but also promotes their personal growth and spiritual exploration. Despite some limitations, the findings of this study provide new perspectives for understanding the religious engagement and spiritual needs of modern youth and provide evidence for the adaptability and relevance of religious practice in modern society. Through this study, we can see the important role of religious and spiritual practices in youth culture and how these practices can help young people find inner peace and personal meaning. Future research should continue to explore the broad application of these findings and how these practices can be more effectively integrated into the daily lives of young people to promote their overall wellbeing and social adaptation.

## Data Availability

The raw data supporting the conclusions of this article will be made available by the authors, without undue reservation.
